# Mantle transcriptome sequencing of *Mytilus* spp. and identification of putative biomineralization genes

**DOI:** 10.7717/peerj.6245

**Published:** 2019-01-14

**Authors:** Magdalena Malachowicz, Roman Wenne

**Affiliations:** Institute of Oceanology Polish Academy of Sciences, Sopot, Poland

**Keywords:** Transcriptome, Mytilus, Mantle, Biomineralization, Molecular markers, Chitinase, α-Carbonic anhydrase, Tyrosinase

## Abstract

In molluscs, the shell secreted by mantle tissue during the biomineralization process is the first barrier against predators and mechanical damage. Changing environmental conditions, such as ocean acidification, influence shell strength and thus protection of the soft body within. Mussels are marine bivalves with important commercial and ecological value worldwide. Despite this importance, the proteins involved in the biomineralization and pigmentation processes in *Mytilus* spp. remain unclear, as does taxonomy of *Mytilus* taxa, though there have been many molecular studies. To further understanding in these areas, this study aimed to characterize and compare mantle transcriptomes of four mussel taxa using next generation sequencing. Mussels representing four taxa, were collected from several localities and RNA from mantle tissue was extracted. RNA sequences obtained were assembled, annotated and potential molecular markers, including simple sequence repeats (SSRs) and single nucleotide polymorphisms (SNPs) were identified. Candidate contigs putatively related to biomineralization and pigmentation processes were then selected and several transcripts were chosen for phylogenetic analyses from the Bivalvia class. Transcriptome comparisons between *Mytilus* taxa, including gene ontology (GO) enrichment analysis and orthologues identification were performed. Of assembled contigs, 46.57%, 37.28% and 17.53% were annotated using NCBI NR, GO and Kyoto Encyclopedia of Genes and Genomes databases, respectively. Potential SSRs (483) and SNPs (1,497) were identified. Results presented a total of 1,292 contigs putatively involved in biomineralization and melanogenesis. Phylogenetic analyses of α-carbonic anhydrase, chitinase and tyrosinase revealed complex evolutionary history and diversity of these genes, which may be a result of duplication events or adaptation to different environments in mussels and other bivalves. Enrichment analyses revealed GO terms associated with pH and thermal response in *Mytilus edulis* from the North Sea and *M. galloprovincialis* from the Mediterranean Sea. The phylogenetic analysis within the genus *Mytilus* revealed *M. californianus* and *M. coruscus* to be genetically more distant from the other taxa: *M. trossulus, M. edulis*, *M. chilensis* and *M. galloprovincialis*. This work represents the first mantle transcriptome comparison between *Mytilus* taxa and provides contigs putatively involved in biomineralization.

## Introduction

Mussels of the genus *Mytilus* are widespread in both the Northern and Southern Hemispheres and are important components of coastal ecosystems (Zbawicka et al., 2012; Smietanka et al., 2013; Lockwood, Connor & Gracey, 2015). Mussels are a bivalve species of significant ecological and commercial value worldwide with annual production of 1.2M tonnes (Gosling, 2003; Kijewski et al., 2009; Zbawicka, Trucco & Wenne, 2018). Due to their filter-feeding abilities and sessility, they also play an important role as biosensors for pollution and other environmental changes (including ocean acidification) in coastal areas (De Zwaan & Eertman, 1996; Fitzer et al., 2014). Rising levels of atmospheric CO_2_ affects the ability of molluscs to incorporate calcium carbonate, which is essential for building and maintaining shells (Vendrami et al., 2016). In molluscs biocalcitic structures fulfil a wide range of functions such as protecting the inner soft body from predators and environmental damage, detoxification, and attachment of muscles (Lowenstam, Weiner & Veis, 1990). The shell is an exoskeleton made up of a mineral phase (calcium carbonate, CaCO_3_ ∼95%) and organic macromolecules: proteins, glycoproteins, polysaccharides and lipids (Lowenstam, Weiner & Veis, 1990). Despite being a minor component (<5%), proteins in the shell, referred to as shell matrix proteins (SMPs), may play a key role in biomineralization including size regulation, nucleation, morphology and organization of the growing of CaCO_3_ crystals (Marin et al., 2008).

Shells of bivalves differ in color, shape and marking, according to environmental conditions, for example the shell of the *Mytilus edulis* Linnaeus, 1758 in the intertidal zone is blue–black and heavy, while in the sublittoral region, where mussels are continuously immersed, it is thin and brown (Gosling, 2003). Shell morphology and thickness of various mussels from the genus *Mytilus* have also been associated with variability of environmental conditions, such as sediment types, trophic conditions, wave impact (Steffani & Branch, 2003; Moschino et al., 2015; Telesca et al., 2018).

The mussel shell is formed by the heavy fold mantle tissue, which encloses the internal organs of the animal (Gosling, 2003). The mantle is a complex structure underneath the shell, which consists of connective tissue with hemolymph, neural tissue, muscles and gonads (Gosling, 2003). Moreover, it has a sensory function and plays a role in directing particles onto the gills and in deflecting heavier material, storage of nutrient reserves, in bioaccumulation of metals and other compounds and in shell repair and growth (Gosling, 2003). To identify candidate biomineralization genes, over a dozen mantle transcriptomes have been assembled for mussels (Freer et al., 2014; Bjärnmark et al., 2016; Yarra et al., 2016), clams (Clark et al., 2010; Arivalagan et al., 2016; Sleight et al., 2016), scallops (Shi et al., 2013; Sun et al., 2015) and oysters (Fang et al., 2011; Li et al., 2017a). However, there remains a lack of mantle tissue transcriptome from *M. trossulus* Gould, 1850 and *M. chilensis* Hupé, 1854 as well as candidate biomineralization transcripts, and their comparative analysis within the genus *Mytilus*. In this study, the mantle transcriptomes of the four mussel taxa: *M. edulis*, *M. galloprovincialis* Lamarck, 1819—the Mediterranean Sea and Tasmania, *M. trossulus*, *M. chilensis*, were assembled and characterized using a Roche GS-FLX 454 pyrosequencing system. The aim of the present study was twofold: (1) to identify transcripts putatively involved in biomineralization and melanogenesis, and (2) to perform a comparative transcriptome analysis among different taxa of the genus *Mytilus* based on mantle tissue.

A large number of proteins putatively involved in biomineralization and pigmentation processes were identified in different *Mytilus* taxa. This study is the first to present the mantle tissue transcriptome in *M. trossulus* and *M. chilensis*. The generated data (accession number PRJNA419475) add to the growing sequence database for mussels and provide the first comparison of the mantle tissue using mussel transcriptomes from different taxa.

## Materials and Methods

### RNA extraction and pyrosequencing

Five populations of mussels (*Mytilus* spp.) representing four taxa were collected from several localities: *M. edulis* (North Sea, EduN), *M. trossulus* (Vancouver, TroV), *M. galloprovincialis* (Mediterranean Sea, GalM), *M. galloprovincialis* (Tasmania, GalT), *M. chilensis* (Chile, Chil) (Table 1). All taxa had been identified by single nucleotide polymorphism (SNP) genotyping (Zbawicka et al., 2012; Wenne et al., 2016; Larraín et al., 2017). Specimens chosen for RNA extraction were adults of indeterminate sex, spent. Tissue sampling was standardized across individuals: extraction of total RNA from the central and outer mantle tissue from right and left mussel side was performed using a GenElute Mammalian Total RNA Miniprep Kit (Sigma-Aldrich, St. Louis, MO, USA) according to the manufacturer’s protocol, with minor modifications. Homogenization was carried out in a custom buffer composed of 100 mM Tris, 1.4M NaCl, 20 mM EDTA, 2% CTAB, proteinase K (20 mg/ml) and 0.03 mM 2-mercaptoethanol (Malachowicz, Wenne & Burzynski, 2017). Samples were stored at −20 °C. RNA samples from two to four individuals were pooled for each group in equal proportions to make a total of five libraries. The quantity and quality of total RNA was determined at 260 nm on microplate using an Epoch Microplate Spectrophotometer (BioTek Instruments, Incorporated, Winooski, VT, USA) and gel electrophoresis. RNA integrity was checked using an Agilent 2100 Bioanalyzer (Agilent Technologies, Santa Clara, CA, USA). cDNA libraries were prepared using a cDNA Rapid Library Preparation Kit (Roche Applied Science, Basel, Switzerland) and pyrosequencing was performed by Macrogen Incorporation (Seoul, Korea), using Roche GS-FLX sequencing system (Roche, Basel, Switzerland) according to manufacturer’s instructions.

**Table 1:** Information about mussel (*Mytilus* spp.) samples included in the present study.

Group/library name	Species	Location	Year of sampling	*N*
EduN	*M. edulis*	The North Sea: The Oosterschelde estuary	2006	2
TroV	*M. trossulus*	Canada: Vancouver	2012	2
GalM	*M. galloprovincialis*	The Mediterranean Sea: Trieste and Chioggia and the Black Sea	2012	4
GalT	*M. galloprovincialis*	Australia: Tasmania, Spring Bay	2012	2
Chil	*M. chilensis*	Chile: Punta Arenas and Concepción	2012	2

**Note:**

*N*, number of specimens.

### De novo assembly, annotation, SSR and SNP discovery

All reads from the mantle tissue of five *Mytilus* groups were processed separately, through the same pipeline. Raw reads were pre-processed by removing adapters and low quality sequences (quality score = 0.05, discard reads <50 bp) with CLC Genomics Workbench software (v.7.5.5, CLC Bio, Qiagen, Aarhus, Denmark). Quality reads were assembled into contigs using de-Bruijn graphs in CLC Genomics Workbench with default parameters (Malachowicz, Kijewska & Wenne, 2015; Malachowicz, Wenne & Burzynski, 2017). Contigs were first searched against proteins from the NCBI non-redundant (NR) database using a BLASTX tool implemented in BLAST+ (v.2.2.29) (Altschul et al., 1990), with an *E*-value threshold of 10^−5^ and other default parameters. A list of the top BLAST hits was achieved by using bit score (12th column) and *E*-value values (11th column) with the code: sort -k1,1 -k12,12 nr -k11,11 g -k3,3 gr blastout.txt sort -u -k1,1–merge > besthit. For functional annotation, gene ontology (GO) terms and Kyoto Encyclopedia of Genes and Genomes (KEGG) pathways were assigned to the transcripts using Blast2GO software (Conesa et al., 2005) with *E*-value Hit-Filter = 1.0 *E*^−10^ (other default parameters) and the single directional best-hit method in the KEGG Automatic Annotation Server (Moriya et al., 2007), respectively.

The software, MSDB (Du et al., 2013) was used to discover potential microsatellite markers. Di- to hexa- nucleotide motifs, with minimum thresholds of 6-5-4-4-4 repeats, respectively and 50 bp of flanking sequence length were identified. SNP analyses were carried out using CLC Genomics Workbench. For each transcriptome, clean reads were mapped back to corresponding contigs with an overlap criterion of 70% and a similarity of 90%. Candidate variants were called with the following minimum value parameters: neighborhood quality = 15; quality of central bases = 20; coverage = 20; count = 8; variant frequency = 35.0.

### Identification of potential biomineralization and melanogenesis-related contigs

Candidate contigs related to biomineralization and melanogenesis were identified by searching top BLASTX hits with key words from a reference list of previously reported candidate biomineralization genes (Table S1). Melanogenesis-related transcripts were identified based on KEGG pathways analysis.

Several biomineralization proteins were selected for additional analysis: carbonic anhydrase (CA), chitinase (CHIT), tyrosinase (TYR), dermatopontin (DPT), arginine kinase (AK) and glyceraldehyde-3-phosphate dehydrogenase (GAPDH). Proteins of selected sequences were predicted using OrfPredictor (Min et al., 2005). Functional domains and motifs were identified using Simple Modular Architecture Research Tool (SMART) software, allowing identification and annotation of domain architectures (http://smart.embl-heidelberg.de) (Letunic, Doerks & Bork, 2015). To further scrutinize and compare selected contigs, representative reference bivalve databases were created. Protein sequences described as CA, CHIT, DPT, AK and GAPDH, available at the GenBank database (www.ncbi.nlm.nih.gov) were downloaded (217, 155, 8, 43, 24 sequences, respectively). Only sequences with annotated domains, were selected (199, 123, 8, 43, 24 sequences, respectively). Following this, mussel contigs from the present study were searched against the created databases with an *E*-value threshold of 10^−15^. For phylogenetic reconstruction of *Mytilus* spp. TYR proteins, contigs from this study, amino acid sequences from GenBank (Acc. No: AKS48166, ALA16023, OPL3388), and sequences from previous studies (Aguilera, McDougall & Degnan, 2014; Moreira et al., 2015; Hüning et al., 2016; Sleight et al., 2016) were used. Homologous sequences were aligned using multiple alignment program MUSCLE (Edgar, 2004) with default parameters, and trimmed using TrimAl (Capella-Gutierrez, Silla-Martinez & Gabaldon, 2009) with an 80% threshold. Best substitution models were inferred using ModelFinder (Kalyaanamoorthy et al., 2017), as follow: CA—the Le Gascuel (LG) model (Le & Gascuel, 2008), gamma distribution, four discrete rate categories (+G4); GH18—the Whelan and Goldman (WAG) model (Whelan & Goldman, 2001) + G4 with invariant sites (+I); AK and GAPDH—WAG+ G4; DPT—the Block substitution matrices (Blosum62) model (Henikoff & Henikoff, 1992) + G4, with frequencies (+F); TYR—Blosum62+F+I+G4 model. Three phylogenetic models were run for each alignment. Neighbor-joining (NJ) trees were performed using MEGA X (Kumar et al., 2018) and the Jones–Taylor–Thornton (JTT) substitution model (Jones, Taylor & Thornton, 1992). Maximum likelihood reconstructions were performed using IQ-TREE (Nguyen et al., 2015) with 1,000 bootstrap replicates. Bayesian inferences were constructed using MrBayes v.3.2 (Ronquist et al., 2012) with parameters: ngen = 1,500,000; samplefreq = 100; printfreq = 100; nchain = 4; starttree = random. Trees were visualized and edited using MEGA X and manually combined to produce a consensus tree for each protein alignment.

### Comparative transcriptomic analysis

Homologous transcripts present in all five groups were identified by using bi-directional tBLASTx analyses conducted between each transcriptome with an *E*-value cut-off of 10^−15^. A list of top BLAST hits was achieved by using bit score (12th column) and *E*-value values (11th column) with the code: sort -k1, 1 -k12, 12 nr -k11, 11 g -k3,3 gr blastout.txt | sort -u -k1,1–merge>besthit. Further, results from this study were compared with available *Mytilus* spp. mantle sequences. The accession numbers are: for *M. edulis*: PRJEB4516 (Freer et al., 2014), PRJNA30561 (Yarra et al., 2016); for *M. galloprovincialis*: PRJNA230138 (Moreira et al., 2015), PRJNA295512 (Bjärnmark et al., 2016). For this purpose, a de novo transcriptome assembly was performed using the raw reads deposited in the NCBI Sequence Read Archive (SRA). The Illumina HiSeq raw reads were filtered using Trimmomatic v.0.32 (Bolger, Lohse & Usadel, 2014) and retained reads were assembled using Trinity program with default parameters (Grabherr et al., 2011).

To find enriched GO terms between groups, Fisher’s exact test was performed with Blast2GO software, applying a one-tailed test, removing double IDs, with a false discovery rate (FDR) cut-off of 0.05. As a test-set, list of contigs from each group was used. As a background (reference-set), a fusion of the five transcriptomes was used.

To identify putative orthologs among the genus of *Mytilus*, transcripts from this study and from *M. coruscus* (Xu et al., 2016), *M. californianus* (Romiguier et al., 2014) were analyzed using OrthoMCL software with default settings (Li, Stoeckert & Roos, 2003). The putative orthologous clusters were aligned with a MUSCLE tool and then concatenated in CLC Genomics Workbench. A splits network was created with SplitsTree 4.13.1 (Huson & Bryant, 2006), using the neighbor-net algorithm under pdistance model. A phylogenetic tree was inferred using a NJ model in MEGA X, with the JTT + G4 substitution model and 1,000 bootstrap replicates.

## Results

### Assembly, annotation, SSR and SNP detection

A total of 753,135 raw reads with an average read length of 342 bp were generated from sequencing of the *Mytilus* spp. mantle transcriptomes. After filtering, 743,773 (98.75%) high-quality reads were used for de novo assembly. In total, 20,982 contigs with an average length of 529 bp were obtained (Table S2). Mussel raw sequences from this study were deposited in NCBI SRA under accession number PRJNA419475.

BLAST analysis against NCBI NR database showed that between 43.19% (TroV) and 52.18% (GalM) of transcripts had a significant hit (Tables S2 and S3). As expected, most of the annotated transcripts were homologous to proteins from the Mollusca phylum: Pacific oyster (*C. gigas*, av. 50%), *Mytilus* spp. (av. 10%) and owl limpet (*L. gigantea*, av. 5%) (Fig. S1). KEGG analysis showed that a total of 3,679 (17.53%) transcripts were assigned to 271 biochemical pathways. The number of sequences assigned to each pathway was different across groups (Table S2). However, the most represented pathways in all groups included focal adhesion, phagosome, ribosome and apoptosis (Table S4). The signal transduction, endocrine system, immune system and cellular community were the most represented KO (KEGG orthology) subcategories in all groups (Fig. S2). In total, 35,383 GO terms were assigned to 7,824 (37.28%) contigs (Table S2). The main GO category distribution was similar in all *Mytilus* spp. groups, the dominant was the biological process category (BP), followed by cellular component and molecular function (MF) (Fig. S3). In this study, 483 potential simple sequence repeats (SSRs) were identified in 645 contigs (3.07% of the total number of contigs) (Table S5A). Among the identified putative markers, the most highly represented in the EduN, TroV, GalM and GalT groups were di-nucleotide repeats, and tri-nucleotide in Chil. AT was the dominating motif in all groups (Table S5B). In addition, a total of 1,497 putative SNPs were detected in all groups. The A/G variation type was dominant in EduN and TroV groups, whereas in other groups the C/T was dominant (Fig. S4).

### Contigs potentially involved in biomineralization and melanogenesis

BLASTx searching revealed 1,292 contigs with significant sequence similarity to candidate biomineralization proteins (Table S6). SMPs such as, nacrein, calmodulin, collagens, perlucin, perlustrin, PIF were found among identified contigs (see Table S6). Transcripts involved in ion and acid-base regulation, protein homeostasis and anti-oxidation were also found including V-type proton ATPases, sodium/potassium-transporting ATPases, heat shock 70 kDa protein (HSP70) and glutathione peroxidase. Other annotations included C1q-domain-containing protein, cAMP-response element-binding protein and cold-shock protein (CSP) (Table S6). Of annotated biomineralization contigs, 68.65% had domains, including chitin binding, EF hand, sushi, Von Willebrand factor A (Table S6).

**Figure 1:**
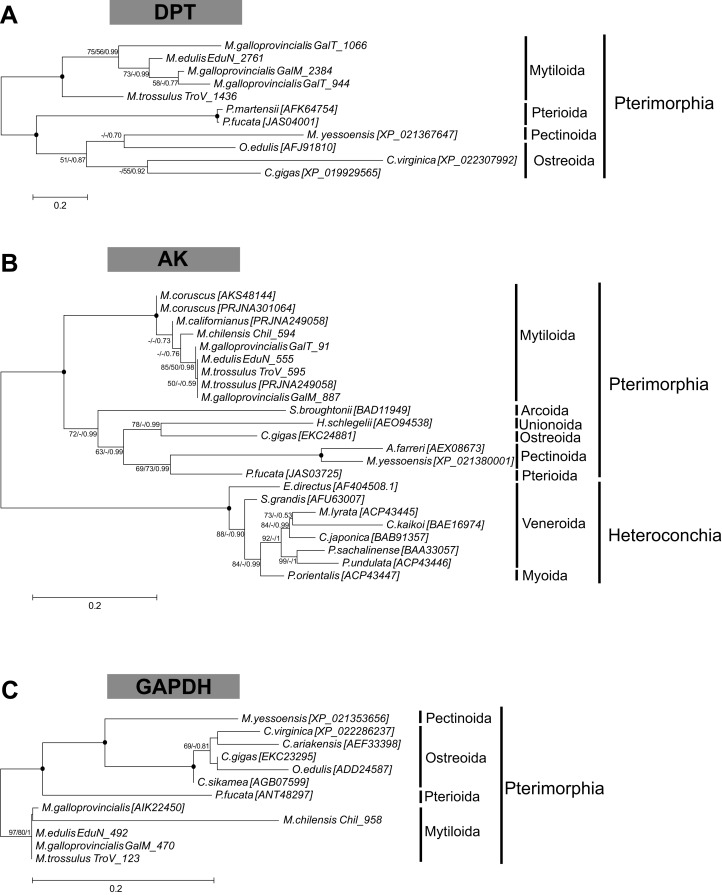
Molecular phylogenetic analysis of dermatopontin (A), arginine kinase (B) and glyceraldehyde-3-phosphate dehydrogenase (C) protein sequences in Bivalvia class. A consensus tree based on maximum likelihood (ML) topology. The numbers of the nodes show the bootstrap values (BV) >50% and Bayesian posterior probabilities (BPP) >0.50, as follows: ML BV/NJ BV/BIs BPP. A black dot at the node represents BV >90% and BPP >0.90.

**Figure 2:**
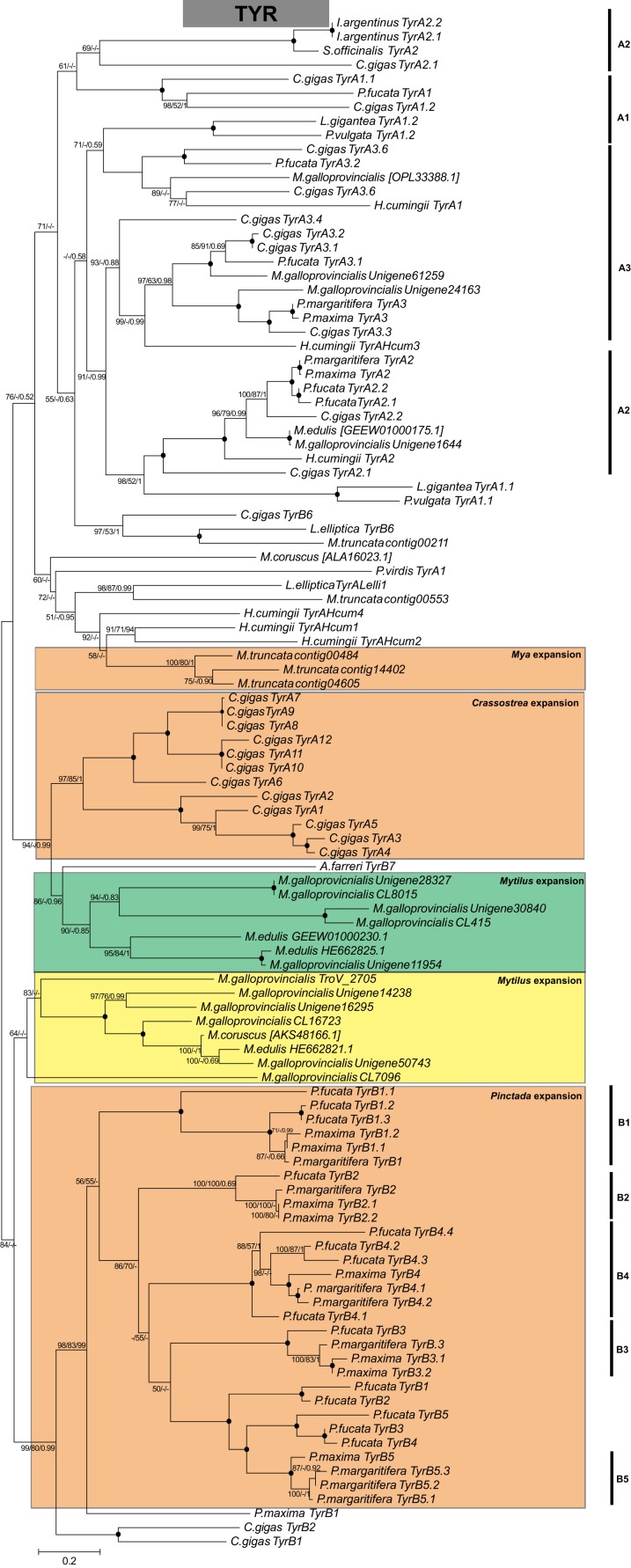
Molecular phylogenetic analysis of tyrosinase (TYR) protein sequences in Bivalvia class. A consensus tree based on maximum likelihood (ML) topology. The numbers of the nodes show the bootstrap values (BV) >50% and Bayesian posterior probabilities (BPP) >0.50, as follows: ML BV/NJ BV/BIs BPP. A black dot at the node represents BV >90% and BPP >0.90. Bivalve TyrA ortholog groups are annotated as A1–A3, *Pinctada* spp. TyrB ortholog groups are annotated as B1–B5. Color code: green and yellow—two possible *Mytilus* expansions; orange—*Crassostrea*, *Mya*, *Pinctada* expansions, described in previous studies (Aguilera, McDougall & Degnan, 2014; Sleight et al., 2016).

**Figure 3:**
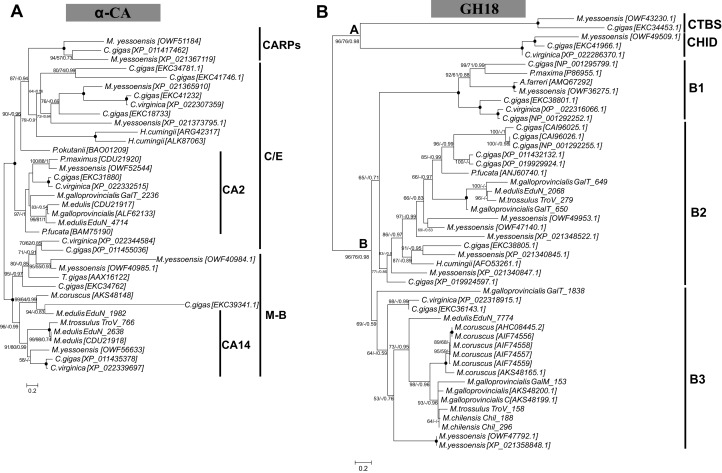
Molecular phylogenetic analysis of α-carbonic anhydrase (A) and glycoside hydrolase family 18 (B) protein sequences in Bivalvia class. A consensus tree based on maximum likelihood (ML) topology. The numbers of the nodes show the bootstrap values (BV) >50% and Bayesian posterior probabilities (BPP) >0.50, as follows: ML BV/NJ BV/BIs BPP. A black dot at the node represents BV >90% and BPP >0.90. Abbreviations: CARPs, carbonic anhydrase related proteins; C/E, cytosolic/extracellular; M-B, membrane-bound.

Several biomineralization proteins were selected for more comprehensive analyses. In *M. galloprovincialis*, *M. edulis* and *M. trossulus* groups, five contigs (GalT_1066, GalT_1436, EduN_2761, GalM_2384, TroV_1436) were identified as DPT with DERM domain. A phylogenetic analysis of DPT proteins in Bivalvia generated a separate, well-supported clade of Mytiloida members, suggesting that these contigs are *Mytilus* spp. orthologues of DPT (Fig. 1A). Contigs with 81–95% sequence identity to AK were identified in all groups. The phylogenetic analysis showed two separate clades of Pterimorphia and Heteroconchia subclasses (Fig. 1B). Contigs Chil_958, EduN_492, GalM_470 and TroV_123 encoded proteins with similarity to GAPDH and possessed the Gp_dh_C domain. Alignment with other known GAPDHs from Ostreoida, Pectinoida and Pterioida members revealed many conserved regions among these species (Fig. S5). However, a phylogenetic analysis showed two well-supported separate clades of Mytiloida and Ostreoida with Pectinoida orders (Fig. 1C). TYR amino acid sequences derived were aligned with available molluscan TYR sequences. A phylogenetic analysis revealed that mussel TYR, in this study, created two separate clades: clade A and clade B (Fig. 2), described earlier as ancestral and bivalve-specific (Aguilera, McDougall & Degnan, 2014). It is possible that there were two small expansions within *Mytilus*, which is a common feature in bivalves. In *M. galloprovincialis* there were TyrA2, TyrA3 orthologues shared with the genera *Crassostrea* and *Pinctada*, indicating gene duplication before the divergence of lineages A and B. Searches in mussel transcriptomes revealed five contigs (in EduN, TroV and GalT groups) that pertain to the CA gene family and 10 contigs (in all transcriptomes) that showed similarity to the glycoside hydrolase family 18 (GH18) CHIT members (Table 2). A SMART analysis revealed CA domains in annotated CA sequences, and GH18_CHIT-like superfamily domain in GH18 sequences. The chitin binding peritrophin-A domain (CBM_14 superfamily) was found in transcripts recognized as acidic mammalian CHIT (GalT_650, GalT_649 and TroV_158). The phylogenetic analysis of CA in the Bivalvia class revealed three major clusters: α-carbonic anhydrases related proteins (α-CARPs), cytosolic/extracellular α-carbonic anhydrases (α-CAs) and membrane-bound α-CAs. There were two visible sub-clusters: CA2 and CA14 (Fig. 3A). Two major clades, A and B were resolved in the GH18 CHIT family phylogenetic tree. The A cluster consisted of CHIT domain-containing proteins (CHIDs, with GH18_SI-CLP domain), and Di-N-acetylchitobiases. In contrast to A, cluster B contained genes involved in chitin degradation (CHIT and CHIT-like proteins), creating three sub-clades (B1, B2, B3) (Fig. 3B). Presented phylogenetic reconstructions were supported by the high values of the nodes.

**Table 2:** α-CA and GH18 proteins identified in mantle transcriptomes, according to BLASTX searches.

Contig	Size (nt)	Acc. number (NCBI)	Homology in NCBI (NR) database		
			Description	NCBI Acc. code	*E*-value
EduN_1982	697	MG827120	*M. coruscus* carbonic anhydrase-like protein	AKS48148.1	1*E*-46
EduN_2638	1,264	MG827121	*C. virginica* carbonic anhydrase 14-like isoform X3	XP_022339697.1	8*E*-81
EduN_4714	404	MG827122	*M. galloprovincialis* carbonic anhydrase II	ALF62133.1	3*E*-65
TroV_766	1,475	MG827123	*C. gigas* carbonic anhydrase 14-like isoform X2	XP_011435377.1	2*E*-59
GalT_2236	347	MG827124	*C. gigas* carbonic anhydrase 2-like	XP_011429671.1	5*E*-40
EduN_7774	362	MG827131	*M. yessoensis* chitinase 3	OWF51745.1	7*E*-37
EduN_2068	1,194	MG827125	*M. yessoensis* Acidic mammalian chitinase	OWF47140.1	1*E*-52
TroV_279	1,678	MG827132	*M. yessoensis* Acidic mammalian chitinase	OWF47140.1	4*E*-62
TroV_158	1,647	MG827133	*M. coruscus* mytchitin 5	AIF74559.1	0.00
GalM_153	195	MG827126	*M. coruscus* mytchitin 4	AIF74558.1	2*E*-25
Chil_296	1,026	MG827134	*M. coruscus* chitinase-like protein-1	AKS48165.1	8*E*-157
Chil_188	500	MG827127	*M. galloprovincialis* chitinase-like protein-3	AKS48199.1	9*E*-88
GalT_650	2,725	MG827128	*M. yessoensis* Acidic mammalian chitinase	OWF47140.1	3*E*-122
GalT_649	705	MG827129	*M. yessoensis* Acidic mammalian chitinase	OWF47140.1	1*E*-36
GalT_1838	847	MG827130	*M. yessoensis* probable chitinase 10	XP_021358847.1	2*E*-33

A total of 42, 99, 19 and 62 contigs from the mussel mantle transcriptomes were assigned to 13, 40, 8 and 15 KO terms in melanogenesis, endocytosis, TYR and calcium signaling KEGG pathways, respectively (Table S7).

### Comparative transcriptomics

Bi-directional Best Hit (BBH) method was used to identify 6,130 homologous pairs between transcriptomes of which 552 were shared between all five groups (Fig. S6). The highest number of homologs were found between pairs: EduN_TroV, EduN_GalT and EduN_GalM (1,029, 946, 818, respectively). Putative homologous pairs were ordered according to sequence similarity of BLAST alignments (Fig. S6). In general most homologous pairs exhibited a sequence similarity of 90–100%. The highest similarity per no. of homologs was found between EduN_GalM (85.45%), followed by EduN_Chil (84.89%), GalM_Chil (84.55%), GalM_GalT (84.12%). The lowest similarity to other groups was shown by TroV. Transcriptomes obtained from this study were compared to available mantle sequences for *M. edulis* and *M. galloprovincialis* using tBLASTx. In total, 18,456 (87.96%) contigs from this study were homologous with one of the four created mantle transcriptomes, but 12.04% might represent new sequence information. Additionally, between 47.8% and 81.5% of homologous contigs had high similarity (>90%; Fig. S7).

For more accurate analysis, Fisher’s exact test was applied to discover significantly over-represented GO terms in each group. The most interesting GO enrichments associated with BP and MF were identified only in EduN and GalM groups. The results were reduced to the most specific terms (FDR = 0.01) (Figs. S8 and S9). A total of 25 GO terms were enriched in the *M. edulis* from the North Sea including cellular response to chemical stimulus (GO:0070887), positive regulation of cellular process (GO:0048522) in the BP category, ATP binding (GO:0005524), calcium ion binding (GO:0005509) in the MF category (Fig. S8). A total of 46 terms were enriched in the *M. galloprovincialis* from the Mediterranean Sea such as organonitrogen compound biosynthetic process (GO:1901566), regulation of biological quality (GO:0065008) in the BP category, metal ion binding (GO:0046872), purine ribonucleotide binding (GO:0032555) in the MF category (Fig. S9).

**Figure 4:**
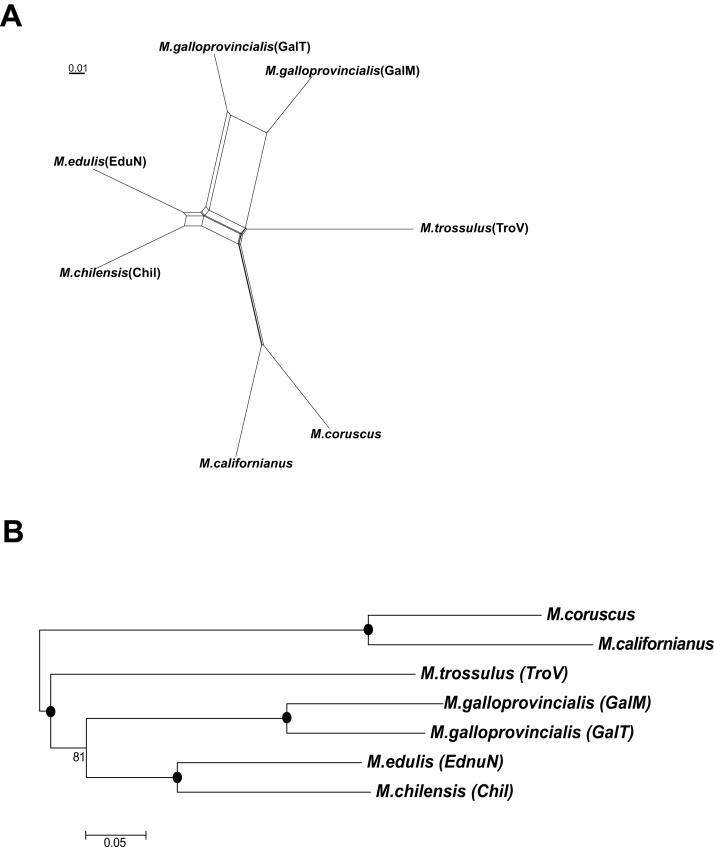
The splits network (A) and phylogeny (B) of six mussels taxa. A black dot at the node represents BV >90%.

Both the neighbor-net splits network (Fig. 4A) and NJ phylogenetic tree (Fig. 4B) showed that *M. chilensis* was the most similar to the *M. edulis* taxon, followed by *M. galloprovincialis* and *M. trossulus*. A separate clade was identified containing *M. californianus* Conrad, 1837 and *M. coruscus* Gould, 1861 (presently accepted as *M. unguiculatus* Valenciennes, 1858) mussels (Fig. 4).

## Discussion

The mollusc shell, formed by calcium carbonate deposition (secreted by the mantle tissue), forms the first barrier against marine predators, mechanical or toxic damage and protects the soft body. This calcified structure is sensitive to environmental conditions: CO_2_ level, temperature, humidity. Moreover, mussels have adapted to different environments that affect the shell formation process and the stability of the secreted composite biomineral (Mann & Jackson, 2014). The sequencing of mantle transcriptome from multiple taxa of *Mytilus* spp. has added to the growing transcriptome resources for mussels and has provided contigs putatively involved in biomineralization. High-quality reads were assembled totaling 20,982 contigs, 9,773 (46.57%) of them were annotated by BLAST searches against NCBI NR protein database (Tables S2 and S3). The top hits showed homology with other bivalves and most annotations were extracted from *C. gigas* Thunberg, 1793 (Fig. S1), as expected due to the availability of an oyster fully sequenced genome. Transcriptome-based markers are a valuable resource for determining functional genetic variation (Malachowicz, Wenne & Burzynski, 2017). In this study, 483 potential SSRs and 1,497 putative mantle-related SNPs were identified. SSRs (Vidal et al., 2009) and SNPs (Vendrami et al., 2016) have previously been developed in mussels using the transcriptome approach, however, this study aimed to identify molecular markers in mantle tissue from several *Mytilus* taxa. These results might be a resource for molecular marker studies associating candidate biomineralization genes with different environmental conditions and/or taxa.

Both GO and KEGG analyses showed a wide array of categories and pathways potentially involved in biomineralization and pigmentation mechanisms (Tables S4 and S7). In all groups, cellular processes: focal adhesion, tight junction, adherens junction and signaling pathways: insulin, calcium, chemokine, ErbR, MAPK and VEGF were identified (Table S4). The importance of these pathways in shell formation has been proposed by Wang et al. (2013) and Liu et al. (2015). Tyrosine metabolism and melanogenesis pathways detected in the mantle transcriptomes presented here might play a fundamental role in the shell pigmentation process. Numerous genes in those pathways were identified such as calmodulin, protein kinase, TYR, β-catenin (Table S7). Moreover, it seems that the Wnt signaling pathway regulation of the transcription factor essential for melanocyte development (MITF) might play a crucial role in melanogenesis (melanocyte development) in bivalves. Another important pathway may be endocytosis, a mechanism for cells to remove nutrients, ligands and to influence pigment granule formation (Feng et al., 2015). Feng et al. (2015) further suggested, that, endocytosis and genes VPS or Rab might have an effect on shell coloration in *Crassostrea gigas* (Thunberg, 1793). These results are in line with previous studies in scallop (Sun et al., 2015) and oysters (Li et al., 2017a).

A total of 1,292 contigs putatively involved in the biomineralization mechanism were also identified. Although most of these transcripts have been reported in *M. edulis* (Hüning et al., 2016) and *M. galloprovincialis* (Gao et al., 2015; Moreira et al., 2015) mantle transcriptomes, new transcripts were identified for *M. trossulus* and *M. chilensis*. This data may contribute to future comparison between different localities of the same taxon, to assess the influence of environment on the shell formation. All presented contigs should be considered as candidate biomineralization proteins since their functions were hypothesized mainly based on expression data, but were not validated (Herlitze et al., 2018). In mussels, the shell is composed of two polymorph forms of calcium carbonate (calcite and aragonite) (Liao et al., 2015). Some matrix proteins, potentially related to the formation of calcite (e.g., TYR) as well as aragonite crystals (e.g., Pif, nacrein) and a number of enzymes metabolizing chitin protein, including CHIT (may be responsible for chitin degradation) and chitin synthase (may be active in construction of the chitin framework) (Feng et al., 2017) were identified in the presented transcriptomes. Perlucin (nucleates growth of CaCO_3_ crystals) (Blank et al., 2003) and calponin (calcium binding protein, may ensure shell elasticity) (Sleight et al., 2016) were also confirmed in the transcriptomes of *Mytilus* spp. by this study (Table S6). In EduN, TroV, GalM and GalT groups, 15 contigs were identified rich in immunoglobulin domains with homology from 34% to 69% sequence identity to hemicentin from *M. yessonis* Jay, 1857, *Crassostrea virginica* Gmelin, 1791 and *C. gigas*. Hemicentin is an extracellular ion-binding protein with an unclear function in shell formation, however, it was previously found in the coral skeletal organic matrix (Ramos-Silva et al., 2013). Another significant protein is chorion peroxidase (TroV_3244) with an unknown role in biomineralization, though it has been suggested that it might be involved in melanogenesis in *S. officinalis* Linnaeus, 1758 (Gesualdo et al., 1997).

In this study, several candidate biomineralization proteins were selected for more comprehensive analysis: DPT, AK and GAPDH, since they were represented in at least four of the five *Mytilus* groups. DPT is an extracellular matrix protein and may participate in nacre formation (Jiao et al., 2012). GAPDH is an enzyme involved in carbohydrate metabolism, which catalyze the formation of 1,3-bisphospho glycerate from glyceraldehyde 3-phosphate and might be involved in the shell growth process (Arivalagan et al., 2016). AK is a protein with ATP-guanido phosphotransferases domain, which may play a role in buffering intracellular pH, important in the extrapallial space with the supersaturation of shell matrix components, including calcium ions (Clark et al., 2010). A phylogenetic reconstruction of these proteins showed a well-supported, separate clade of Mytiloida order in a sister relationship to other Bivalves (Fig. 1). However, the trees presented did not support relationships between the four orders: Mytiloida, Ostreoida, Pterioida and Pectinoida. Only AK phylogeny clearly pictures the relationships among major bivalve lineages Pterimorphia and Heteroconchia, although it should be noted that there is a lack of sequences for DPT and GAPDH proteins. In the case of AK and GAPDH high conservation was observed between *M. edulis, M. galloprovincialis* and *M. trossulus* taxa, suggesting the importance of these candidate biomineralization genes in mussels. Only DPT phylogeny confirmed the expected evolutionary relationships of *M. trossulus*, *M. edulis* and *M. galloprovincialis*.

Rising atmospheric CO_2_ emissions and the direct consequences of it, ocean acidification and ocean warming, have the potential to limit the ability of calcifying marine organisms to produce their protective exoskeleton and shell, and lead to decalcification (Fitzer et al., 2014; Goffredo et al., 2014). Changes in the structural order potentially impact on mussel shell strength, limiting protection from predators and the environment. In order to project the future implications of ocean acidification on marine calcifying organisms the biomineralization mechanism need to be fully understood. Previous studies have explored the impact of acidification on shell formation and on the activity of candidate biomineralization molecules: α-CA, CHIT and TYR in mussels (Hüning et al., 2013; Fitzer et al., 2014). The evolutionary relationships of these genes in metazoan have been previously studied (Aguilera, McDougall & Degnan, 2014; Le Roy et al., 2014; Sleight et al., 2016; Feng et al., 2017), however, there has still been a lack of sequence resources especially from mussels. Given the importance of these genes in biomineralization process, phylogenetic reconstructions using contigs obtained from this study were performed. α-CAs are metalloenzymes which catalyze the reversible hydration of CO_2_ to form bicarbonate ion and proton (Le Roy et al., 2014). In mammals the α-CA family is characterized by 13 different enzymatically active isoforms and three CARPs which appear to lack CA activity (Aspatwar, Tolvanen & Parkkila, 2010). CHIT is hydrolyzing β-1,4-linkages in chitin, which is a major component of the organic matrix in the shell. TYR is a type-3 copper protein superfamily, a multifunctional, shell associated protein, melanocyte-specific marker, potentially involved in pigmentation and essential for the varied coloration of shell in bivalves (Feng et al., 2015; Sun et al., 2015). It has been suggested that TYR represents a protective mechanism against external shell corrosion (Fitzer et al., 2014). This study identified several copies (isoforms) of α-CA, CHIT and TYR proteins in the genus *Mytilus*, which might have been a result of several duplication events and diversification resulting in multiple functionality: immune response, potentially biomineralization and pigmentation (Figs. 2 and 3). This diversity might be also correlated with wide geographical distribution, adaptation to different environments and different selection pressures driving evolution of these specific genes. The contigs identified in this study have provided a new resource for future studies on α-CAs, CHIT and TYR, however, the phylogeny reveals a complex evolutionary history of these genes that requires more data.

To identify homologs and similarity of mantle transcriptomes between different taxa and localizations, BBH method was used. The highest similarity (>80%), was observed between *M. edulis* (EduN), *M. galloprovincialis* (GalM, GalT) and *M. chilensis* (Chil) taxa (Fig. S6). Respectively, 848, 488, 549, 343 and 298 contigs in EduN, TroV, GalM, Chil, GalT groups, did not show homology (*E*-value cut-off 10^−15^) to mantle transcriptomes from previous studies in mussels (Fig. S7). These contigs may represent novel or specific genes for each group or might be related to environment conditions. To further investigate diversity between different *Mytilus* spp. groups, a GO Fisher exact test was carried out. In *M. edulis* from the North Sea (EduN), GO terms related to heat stress response were over-represented, including: apoptotic process, regulation of programmed cell death, cellular protein complex assembly, microtubule-based process and calcium ion binding (Fig. S8). These terms were associated with high pH exposure in the Antarctic pteropod (Johnson & Hofmann, 2016) and thermal response in the genus *Crassostrea* (Zhang et al., 2012, Li et al., 2017b). In *M. galloprovincialis* from the Mediterranean Sea (GalM) metal ion binding, purine ribonucleoside triphosphate binding, lipid transport and oxidative phosphorylation were enriched (Fig. S9). These terms might be associated with pH and temperature response, as found in *P. fucata* Gould, 1850 (Li et al., 2016) and *C. gigas* (Zhang et al., 2012). Thermal and pH response of mantle tissue in both EduN and GalM groups, might be a direct consequence of increasing carbon dioxide (connected with ocean acidification and global warming), which significantly affects marine calcifiers (Li et al., 2016). However, these results should be interpreted with care, since different number of sequences were used to retrieve GO terms.

Over the last decade, understanding of *Mytilus* spp. evolutionary characteristics significantly increased. Several approaches using molecular methods were used, such as molecular markers (Kijewski et al., 2011), SNP genotyping (Gardner et al., 2016) and mitochondrial DNA (Smietanka, Burzynski & Wenne, 2009). In this study phylogenetic relationships were investigated between the six taxa using transcriptome data (Fig. 4). Our analyses showed that *M. edulis*, *M. chilensis*, *M. galloprovincialis* and *M. trossulus* created a separate clade from *M. californianus* and *M. coruscus*, which is consistent with earlier studies of their mitochondrial DNA (Gaitán-Espitia et al., 2016).

## Conclusions

This study investigated mantle transcriptomes of five groups from four *Mytilus* spp. using 454 pyrosequencing technology. Of generated transcripts 46.57% were annotated by BLAST search. Of these 6.16% were contigs potentially involved in biomineralization and pigmentation processes, such as CA, CHIT and TYR. GO enrichment analysis revealed stress response in *M. edulis* (from the North Sea) and *M. galloprovincialis* (from the Mediterranean Sea), which might be related to ocean acidification and global warming. To our knowledge, this study is the first to describe mantle transcriptomes from multiple *Mytilus* spp and provide a basis for further work (using RNA-seq and gene expression patterns) on biomineralization proteins, which may help to further account for mussels diversity and responses to environment conditions.

## Supplemental Information

10.7717/peerj.6245/supp-1 Supplemental Information 1 Characteristics of the homology search of *Mytilus* transcripts. Distribution of an E-value (A) and species annotation (B) based on the NR best hit results.

10.7717/peerj.6245/supp-2 Supplemental Information 2 KEGG analysis of the five *Mytilus* transcriptomes. CP–Cellular Processes, EIP–Environmental Information Processing, GIP–Genetic Information Processing, M–Metabolism, OS–Organismal Systems.

10.7717/peerj.6245/supp-3 Supplemental Information 3 Gene Ontology classification of *Mytilus* transcriptomes: biological process (A), molecular function (B), cellular component (C).

10.7717/peerj.6245/supp-4 Supplemental Information 4 Putative single nucleotide polymorphisms (SNPs) distribution in *Mytilus* spp. mantle transcriptomes.

10.7717/peerj.6245/supp-5 Supplemental Information 5 Alignment of glyceraldehydes-3-phosphate (GAPDH) protein sequences in Bivalvia class.

10.7717/peerj.6245/supp-6 Supplemental Information 6 Sequence similarity distribution of pairwise comparisons between *Mytilus* spp. groups from present study. Color code indicate the percentage of similarity between two groups.

10.7717/peerj.6245/supp-7 Supplemental Information 7 Sequence similarity distribution of pairwise comparisons between *Mytilus* spp. groups: EduN (A), GalM (B), Chil (C), TroV (D), GalT(E) and mantle transcriptomes from *M. edulis* and *M. galloprovincialis*. *M. edulis*: EduG (Freer et al., 2014), EduS (Yarra et al., 2016), *M. galloprovincialis*: GalP (Bjärnmark et al., 2016), GalS (Moreira et al., 2015). Color code indicate the percentage of similarity between two groups.

10.7717/peerj.6245/supp-8 Supplemental Information 8 Over-represented biological process (BP) and molecular function (MF) GO terms in *M. edulis* (EduN).

10.7717/peerj.6245/supp-9 Supplemental Information 9 Over-represented biological process (BP) and molecular function (MF) GO terms in *M. galloprovincialis* (GalM).

10.7717/peerj.6245/supp-10 Supplemental Information 10 A reference list of previously reported candidate biomineralization genes.

10.7717/peerj.6245/supp-11 Supplemental Information 11 Statistical summary of five *Mytilus* transcriptomes.

10.7717/peerj.6245/supp-12 Supplemental Information 12 Table listing *Mytilus* spp. contigs, including description (NCBI NR database), GO and KEGG IDs ascribed to each sequence.

10.7717/peerj.6245/supp-13 Supplemental Information 13 KEGG pathway annotations in mussel transcriptomes.

10.7717/peerj.6245/supp-14 Supplemental Information 14 Summary of simple sequences repeats (SSRs) analysis (A) and distribution based on motif types (B) in the *Mytilus* spp. mantle transcriptomes.

10.7717/peerj.6245/supp-15 Supplemental Information 15 Contigs potentially involved in the biomineralization mechanism identified in mussel transcriptomes, including description, domain ascribed and sequences in FASTA format.

10.7717/peerj.6245/supp-16 Supplemental Information 16 Contigs identified in tyrosinase, endocytosis, calcium signaling and melanogenesis pathways.

10.7717/peerj.6245/supp-17 Supplemental Information 17 α-CA and GH18 sequences with accesion numbers (NCBI) identified in mantle transcriptomes.

## Additional Information and Declarations

### Data Availability

The following information was supplied regarding data availability: The raw data is available on Sequence Read Archive under accession number PRJNA419475. The CA and CHIT sequences described here are accessible via GenBank accession numbers MG827120–MG827134 and are included in Supplemental_Data_File_1. Other sequences are included in Table S6.
